# Rational and Flexible Adaptation of Sentence Production to Ongoing Language Experience

**DOI:** 10.3389/fpsyg.2021.647076

**Published:** 2021-03-25

**Authors:** Malathi Thothathiri

**Affiliations:** Department of Speech, Language and Hearing Sciences, The George Washington University, Washington, DC, United States

**Keywords:** statistical learning, cue validity, executive function, dorsal stream, ventral stream, individual differences, verb bias

## Abstract

Whether sentences are formulated primarily using lexically based or non-lexically based information has been much debated. In this perspective article, I review evidence for rational flexibility in the sentence production architecture. Sentences can be constructed flexibly via lexically dependent or independent routes, and rationally depending on the statistical properties of the input and the validity of lexical vs. abstract cues for predicting sentence structure. Different neural pathways appear to be recruited for individuals with different executive function abilities and for verbs with different statistical properties, suggesting that alternative routes are available for producing the same structure. Together, extant evidence indicates that the human brain adapts to ongoing language experience during adulthood, and that the nature of the adjustment may depend rationally on the statistical contingencies of the current context.

## Introduction

Sentence production involves converting thoughts into structured sequences of words. The representations and processes used to formulate these structured sequences are subject to theoretical debate (see e.g., Konopka and Bock, 2009; Lane and Ferreira, 2010). Consider, for example, a situation where a speaker would like to describe to a listener the information that Amelia had given a bag to John. The speaker's brain could accomplish this communicative act by choosing an abstract structural frame associated with transfer events [e.g., <Agent> <transfer verb> <theme> to <recipient> or Noun-Phrase (NP) Verb (V) NP Preposition NP] and subsequently filling in the specific verb and the other words (e.g., *give, bag*). Alternatively, the structured sequence could be formulated by first choosing the core verb (e.g., *give*) and then accessing the structural information associated with that verb (e.g., where the different arguments of *give* can be placed in a sentence). This debate is often posed as a dichotomy but it is possible that both routes to sentence production are available and can be chosen under different circumstances. The perspective put forth in this paper is that the path to sentence formulation can be rational and flexible i.e., depending on the statistical properties of ongoing language experience, the brain can come to rely on either verb-specific or verb-general representations for sentence production in a given context. This process is rational because the choice is tuned to the statistical contingencies of the current context. It is flexible because the architecture adapts to changing statistical contingencies throughout the lifespan.

Under a rationalist view, learning to understand and produce sentences involves learning which sentence structures are the most likely to be used in the future based on past experience. The human brain can encode and use past experience at different granularities, including all prior input, the most recent input, and input tied to specific cues and contexts (Ellis, 2006). Probability-based tuning is rational because past experience is a good predictor of future occurrence. Additionally, how language is used differs across speakers, dialects, and modalities. Therefore, continual tuning *post-*acquisition allows the language user to adapt appropriately to the current context (Fine et al., 2013). But does sentence formulation adjust rationally and flexibly to ongoing input in this way? Below, I first describe independent evidence for the verb-general and verb-specific routes to sentence production before turning to how the choice between the two adapts to current statistical properties.

Structural priming studies are a predominant source of evidence for the debate between frame-based or abstract syntactic accounts and lexicalist accounts of sentence production. Comprehending or producing a syntactic structure (e.g., a prepositional-object dative like *The wealthy widow gave her Mercedes to the church*) increases the likelihood of speakers using the same structure again with unrelated verbs and nouns (e.g., *The grandfather is reading a story to his grandson*). Such priming, independent of lexical overlap, suggests a role for abstract sentential frames that are not tied to specific lexical items (Bock and Griffin, 2000; Konopka and Bock, 2009; inter alia). Even idiomatic phrases, which are widely assumed to be lexicalized, show abstract priming (Konopka and Bock, 2009). Other non-priming evidence from stem-exchange errors (e.g., “hates the record” becomes “records the hate”) suggests that the production of syntactic-category-consistent stress (e.g., REcord vs. reCORD) is influenced by abstract syntax rather than by lexical selection, consistent with frame-based theories (Lane and Ferreira, 2010).

However, lexical influences on sentence production have also been noted. Structural priming shows a “lexical boost” when the verb repeats between prime and target sentences (Pickering and Branigan, 1998; Hartsuiker et al., 2008). This suggests that structural information tied to specific lexical items can be primed. In naturalistic speech, some verbs (e.g., *give*) can appear in two alternative structures while others are grammatical in only one of the two options [e.g., *donate* is acceptable in prepositional-object (PO) datives like *Laila donated money to the church* but not in double-object (DO) datives like ^*^Laila donated the church money]. Thus, sentence production can be sensitive to the usage pattern of a specific verb (hereafter referred to as “verb bias”).

Earlier evidence had led some researchers to suggest a difference between sentence comprehension and production such that the former is guided more strongly by the lexicon and the latter by abstract syntax (e.g., Arai et al., 2007). However, a recent study compared the two modalities directly and found similar effects, leading the authors to conclude in favor of shared mechanisms for understanding and formulating sentences (Tooley and Bock, 2014). In particular, both abstract structural priming and a lexical boost were detected, indicating that the brain uses structural information stored at lexically independent as well as lexically dependent levels.

If both routes to sentence production are available, how does the brain choose which one to use when? Artificial languages are a useful way to control the language input of participants whose real-life language experiences may be variable. Though these paradigms tap learning a new language, the findings are relevant for natural language use (Wonnacott et al., 2008; Romberg and Saffran, 2010). Further, in the present perspective, language *use* is intricately tied to *learning* the context-appropriate properties of the input. Therefore, I begin by reviewing evidence from artificial language studies before describing the findings for natural language. To preview, this emerging evidence supports the idea of flexibility by showing that:

(1) speakers learn and use new verb biases from short lab-based input sessions not only in an artificial language but also in their native language (Wonnacott et al., 2008; Thothathiri and Rattinger, 2016; Thothathiri et al., 2017. See also Ryskin et al., 2017).
(2) the brain differentially uses alternative processing streams for producing the same structural output for verbs with different statistical properties (Thothathiri and Rattinger, 2015).
(3) frontal executive function regions are recruited differentially in different individuals and for different verb biases (Thothathiri, 2018).

The adaptation appears to be rational, as evidenced by:

(1) sensitivity to verb-specific or verb-general cues depending on the predictive validity of those cues (Thothathiri and Rattinger, 2016; Thothathiri and Braiuca, 2020. See also Perek and Goldberg, 2017).
(2) a division of labor between neural pathways such that effortful semantic processing is engaged only when simpler contingencies are unavailable (Thothathiri and Rattinger, 2015).

### Rational and Flexible Adaptation of Sentence Production in an Artificial Language

In a seminal artificial language study, Wonnacott et al. (2008) showed that adult learners tracked both verb-specific and verb-general statistics and used these sources of information in a rational manner that was dependent on the distribution of verbs and verb types in the input language. Specifically, sentence production after language exposure showed a more lexically specific pattern for high frequency verbs and/or if most verbs in the language were biased toward one or another structure and did not appear in both structures (making individual verbs useful predictors for how they should be used). Conversely, verbs were more likely to be generalized to a structure that they had not appeared in if they were low frequency (providing insufficient verb-specific information) or if the language predominantly contained alternating verbs that appeared in both structures (biasing toward verb-general patterns). The authors concluded that the findings were consistent with a rational Bayesian approach to learning (see also Perfors et al., 2010).

Thothathiri and Rattinger (2016) extended these findings to different types of cues, namely verb-specific syntactic distribution and verb-general semantics-to-structure mappings. They demonstrated that adults could learn which cue was a better predictor of structures heard in the input and prioritize the cue with higher validity for guiding subsequent language use. In Experiment 1, participants were exposed to an artificial language where two alternative structures (Agent-Patient vs. Patient-Agent order) were used equally often to describe transitive actions, making the event semantics non-predictive. Ten out of 12 verbs were biased to appear in one of the two structures, making the verb cue highly predictive of the structure heard during input. Under these conditions, participants' free-choice sentence production in a subsequent test showed a verb-specific pattern, with higher Patient-Agent order produced for verbs that were heard in that order than for verbs that were not. Experiments 2 and 3 (with new participants) made the verb-general semantic cue more predictive than the verb cue by associating two different word orders with two different kinds of events (an event involving an instrument vs. a modifier). Notably, 10 out of 12 verbs were still biased to appear in one of the two structures. Thus, the verb was still highly (but not 100%) predictive. However, the competing semantic cue—namely, whether the observed event involved an instrument or a modifier—was even more (100%) predictive. Under these conditions, speakers overrode verb-specific statistics and used the structure that was appropriate for the event semantics. The authors concluded that sentence production need not be exclusively lexically conservative or generalized. Instead, it can be guided flexibly and rationally by different representations depending on the predictive validities of different cues (Bates and MacWhinney, 1987, 1989; Goldberg et al., 2005; MacWhinney, 2013).

### Rational and Flexible Adaptation of Sentence Production in the Speakers' Native Language

Subsequent studies using a similar methodology in the speakers' native language (English) showed that language users maintain some flexibility in adulthood (Thothathiri et al., 2017; Thothathiri, 2018; Thothathiri and Braiuca, 2020). English speakers learned to use new biases for known dative verbs and a new semantic cue for known dative structures in a manner consistent with cue validity. This is remarkable given the extent of prior English exposure for a speaker who is 18 years or older. The results highlight the fact that language continues to adapt past the childhood stage of acquisition (see also Kamide, 2012; Kroczek and Gunter, 2017; Ryskin et al., 2017) and that the brain rationally learns to use cues that are highly predictive in the current context.

In Thothathiri and colleagues' natural language experiments, participants were provided with lab-based English input containing dative sentences (Thothathiri et al., 2017; Thothathiri, 2018; Thothathiri and Braiuca, 2020). As before, different verbs were biased to appear in different structures, with some appearing exclusively in DO, others exclusively in PO, and yet others equally in both. The assignment of different dative verbs to different bias conditions was counterbalanced across lists. Would native English speakers adapt flexibly to these new biases for known verbs? Thothathiri et al. (2017) found that they did. Across this and other studies below, DO datives were uniformly less common than PO, suggesting that it was the harder structure (note: these DO datives contained full-noun-phrase objects, which occur less commonly in a DO structure than pronouns). Within this overarching tendency, there was differentiation between bias conditions: speakers were most likely to produce DO with verbs that had been heard only in that structure during lab-based exposure and least likely to do so with verbs that had been heard only in the competing PO (with Equi or equal-DO-PO verbs in between), resulting in a significant linear pattern (DO-only > Equi > PO-only).

In a subsequent study, Thothathiri and Braiuca (2020) investigated whether adaptation to new input depends rationally on the relative validity of verb-specific vs. general semantic cues. As before, participants were exposed to lab-based dative input with different verbs assigned to different bias conditions. However, the new experiments included a 100% predictive semantic cue—complete transfer actions where the theme successfully reached the recipient were always described using DO while incomplete transfers were always described using PO. Will event semantics override verb-specific statistics because it has higher cue validity (as in the artificial language experiments in Thothathiri and Rattinger, 2016)? The results presented a nuanced picture. Sentence structure choice and utterance characteristics showed an influence of event semantics when the semantic cue was much more predictive than individual verbs (100 vs. 60 or 70%) but not when the two cues were closer in their validities (100 vs. 90%). In fact, there was a reliable effect of the verb and not the semantic cue in the latter case despite the fact that the verb cue had lower validity. These patterns led the authors to conclude that prior knowledge about the relevance of the verb cue for English datives could mean that it continues to influence native language sentence production under new input conditions. Although the human brain can track and use statistical associations rationally, it is subject to selection biases because some cues might be attended to more selectively and weighted more heavily than is warranted by their predictive validity (see Ellis, 2006 for discussion of similar issues within second language acquisition).

### Neural Mechanisms

Functional magnetic resonance imaging (fMRI) studies provide complementary evidence for rational and flexible adaptation at the level of neural mechanisms. Prior research has suggested that the brain rationally employs “division of labor” between semantic and non-semantic processes for language processing (Plaut et al., 1996; Ueno et al., 2014). In the context of sentence production, the brain flexibly weights the ventral (semantic) and dorsal (non-semantic) streams differently for producing the same dative structure for verbs with different statistical properties. The weightings appear to be rational, favoring effortful semantic processing only when necessary i.e., when there are no easier contingencies present in the input for a given verb.

Thothathiri and Rattinger (2015) first demonstrated flexibility and rational division of labor in an artificial language paradigm. After exposure to the language (as described above), participants' brains were scanned during sentence production in a separate session. The analyses focused on whether producing the harder word order (Patient-Agent) compared to the common one (Agent-Patient) recruited different regions for verbs with different biases (Agent-Patient only, Patient-Agent only, or Equi). The results showed greater bilateral temporal lobe activation and greater functional connectivity between speech motor areas and the right temporal lobe for Equi verbs than for verbs that had appeared in a single order during the input phase. Thus, there was increased involvement of the ventral stream for Equi verbs, which could have resulted from competition between multiple structures for the same verb and deeper semantic processing for identifying meaning-to-order mappings. By contrast, verbs encountered in a single consistent mapping may have been directly associated with their corresponding structures without extensive semantic processing^1^. More broadly, the results showed that the brain can accomplish the same structural output using different alternative pathways.

The brain might also rationally adapt by using different resources in individuals with different cognitive profiles. The relevant studies have focused on frontal-cortex-supported executive function because of its documented role in adaptive, context-appropriate behavior (Koechlin, 2016). Thothathiri and Rattinger (2015) found that better executive function as measured by the Stroop task correlated with a higher proportion of the harder Patient-Agent order for Equi verbs but not for verbs that appeared in a single order. Thus, input statistical properties (verb bias condition) interacted with learner characteristics (Stroop performance) in predicting sentence production choices. This finding was later corroborated by Thothathiri et al. (2017), who examined native language production using English dative structures and found a correlation between individuals' Stroop performance and their production of the harder DO dative for Equi but not for other verbs. A subset of the participants in the latter study took part in a subsequent fMRI session where their brains were scanned during free-choice dative sentence production (Thothathiri, 2018). When producing the harder DO dative after the easier PO dative, participants with better Stroop performance activated the anterior cingulate cortex (ACC) more than those with poorer performance. Furthermore, there was an interaction between learner characteristics and input statistical properties such that individual differences in ACC activation were maximal for PO-only verbs produced in the opposite DO, smallest for DO-only verbs produced in DO, and in between the two for Equi verbs. Functionally, ACC activation was correlated with increased DO production over time for Equi and decreased DO production for PO-only verbs (there was no correlation for DO-only verbs). This suggests that the ACC influences language production in different ways for different verbs in a manner that is consistent with recent experience. It can help boost the production of a difficult sentence structure that is in competition with an easier structure if that structure is sanctioned by recent statistical experience (as in the case of Equi verbs)^2^. Conversely, it can help suppress the production of that same structure if recent experience suggests that the structure is not sanctioned (as for PO-only verbs). Together, these findings raise the intriguing possibility that ACC (and other frontal regions) might be involved in rational and flexible adaptation of language based on speaker, input and context characteristics.

## Discussion

The proposed perspective is consistent with longstanding ideas in the study of language, including cue validity (Bates and MacWhinney, 1987), constraint-based sentence processing (MacDonald et al., 1994; Trueswell and Tanenhaus, 1994), division of labor (Plaut et al., 1996; Ueno et al., 2014), and Bayesian learning (Perfors et al., 2010). The available evidence is intriguing but many open questions remain, which are summarized in Table 1.

**Table 1:** Open questions.

**Open questions for future research**
*Input statistical factors*
(1) What is the effect of prior knowledge about the validities of different cues? Under what conditions, if any, do speakers override prior knowledge? (2) What are the relevant grains of prior knowledge? Does the brain track predictive validities separately for different structural alternations within a language? (3) Are there conditions (e.g., discourse contexts) under which speakers ignore predictive validities entirely? What features might such conditions share?
*Brain regions and mechanisms*
(1) What are the relevant individual differences in cognitive abilities for sentence production? Are these differences and their effects stable over time? (2) What is the division of labor between ventral and dorsal streams for different structures and input conditions? (3) Is executive function necessary or merely facilitative for flexibly choosing between alternative routes to sentence production? (4) What mechanisms are used to consolidate prior and ongoing language experiences?

For example, Thothathiri and Braiuca (2020) suggested that prior knowledge about the relevance of verb bias for English datives could have continued to affect speakers' sentence production in the new context. The nature of the relevant prior knowledge as well as the mechanisms used to consolidate prior and ongoing language experiences remain to be fleshed out (but see Chang et al., 2006; Fine et al., 2013). Multiple studies suggest flexibility in the cues and pathways used for sentence production (Thothathiri and Rattinger, 2015, 2016; Thothathiri, 2018) but additional work is needed to build a comprehensive theoretical framework that explains (a) how predictive validity might rationally change the weighting of different brain regions, and (b) how executive function may be used to select sentence structures under different conditions and for different individuals. Going beyond these questions that are closely related to the perspective described here, it is also important to investigate how context-specific the effects of exposure are and how long they last (Wells et al., 2009; Kamide, 2012).

## Data Availability Statement

The original contributions presented in the study are included in the article/supplementary material, further inquiries can be directed to the corresponding author.

## Ethics Statement

The studies involving human participants were reviewed and approved by the Institutional Review Board at GWU. The patients/participants provided their written informed consent to participate in this study.

## Author Contributions

MT supervised all the research reported here and wrote all versions of the manuscript.

## Conflict of Interest

The author declares that the research was conducted in the absence of any commercial or financial relationships that could be construed as a potential conflict of interest.

## Footnotes

^1^This is analogous to reading aloud regular words, whose letters can be translated directly to the corresponding sounds, without lexical semantic processing.

^2^DO-biased verbs appeared repeatedly and only in the DO structure. This statistical association facilitates DO production for these verbs without much competition from the alternative PO structure.
